# Perception of Overall Health, Weight Status, and Gaining Weight in Relationship With Self-Reported BMI Among High School Students

**DOI:** 10.7759/cureus.19637

**Published:** 2021-11-16

**Authors:** Deepesh Khanna, Cody M Mutter, Payal Kahar

**Affiliations:** 1 Foundational Sciences, Nova Southeastern University Dr. Kiran C. Patel College of Osteopathic Medicine, Clearwater, USA; 2 Basic Sciences, Nova Southeastern University Dr. Kiran C. Patel College of Osteopathic Medicine, Clearwater, USA; 3 Health Sciences, Florida Gulf Coast University, Fort Myers, USA

**Keywords:** self-perceived overall health, high school students, body image, healthy lifestyle, overweight, underweight, weight gain, body mass index: bmi, weight status, health

## Abstract

Background: Correct perception of weight status and gaining weight are important motivational factors for physical activity among overweight and obese children. However, misperception is common.

Objective: The objective of this study is to assess perceptions of overall health, weight status, and weight gain in relation to BMI among high school students.

Methods: A face-to-face validated survey-based study was conducted among high school students. BMI was calculated based on the self-reported height and weight to compare with perceived weight status based on a question: “Do you consider yourself to be underweight, overweight, about right, or obese?" Participants were asked to rate their health and how much they worry about gaining weight. Descriptive and chi-square tests were used for analysis. The level of significance was 0.05.

Results: The results of this study show female students are more likely to perceive themselves as overweight and worried about gaining weight compared to their male peers. The results also show that a low percentage of male and female students rate their overall health as poor with an overweight BMI.

Conclusion: The results of this study provide the framework for understanding the differences in how male and female high school students perceive their health, weight status, and weight gain in relation to BMI. Inaccurate perception of one’s weight status increases the risk of being overweight/obese and decreases the likelihood that students will engage in healthy lifestyle behaviors.

## Introduction

Obesity has been and continues to be a major public health crisis, not only in the United States (US) but on a global scale. [1] The increasing rates of obesity over the past 50 years among adults are alarming [2,3]. The rates of overweight and obese children are also on the rise and the increasing prevalence of early chronic disease onset such as type II diabetes in children [1,3,4]. Since the year 1980, the worldwide prevalence of obesity has doubled, resulting in nearly one-third of the entire adult global population being classified as overweight or obese [1]. As of 2015, it was estimated that roughly 107.7 million children and 603.7 million adults were considered obese [1]. Currently, obesity affects nearly 93.3 million adults, and 18.5% of children in the US or roughly 13.7 million children [3,4]. Linear time trend forecasts suggest that by the year 2030 approximately 51% of adults in the US will be obese, equating to nearly $549.5 billion in additional medical expenditures compared to if obesity rates were to remain at the 2010 rates in 2030 [5]. As rates of obesity in adults continue to climb, similar trends in children will follow, especially in children that have obese parents. For example, it has been shown that obesity in one parent increases the risk of obesity in their child by two to three times and up to 15 times if both parents are obese [6]. It should be noted that children with obesity that persists into adulthood have a significantly increased risk of type II diabetes, hypertension, dyslipidemia, depression or depression-like symptoms, and carotid-artery atherosclerosis compared to adults who have never been afflicted with obesity [6-10].

A major consequence of obesity in adolescents includes various psychosocial comorbidities such as anxiety, depression, low self-esteem, dissatisfaction with body image, bullying, and lower quality of life [10,11]. Research showed that body image is of particular importance in adolescence and having a negative perception of one’s own body has been shown to yield a wide array of psychological stressors [12]. Previous work has shown that nutritional behaviors and body weight status of adolescents depend on how they perceive their weight as well as how their peers perceive their weight, but no correlation was shown regarding their parents’ perception [13]. It has also been shown in a sample of White adolescent females in Pennsylvania that family, friends, and various forms of media contribute to the development of weight control behavior [14]. From this study, it was observed that media sensitivity played the most significant role (p<0.0001) in membership to a weight-control group compared to a non-dieter group [14]. Due to the previous study having been conducted solely on White adolescent females, it should be noted that additional factors are pertinent to obesity in minority groups such as socioeconomic status and access to healthcare, these topics are beyond the scope of this study but should not be ignored. Skewed perception of weight status relative to self-reported or measured BMI has been shown to be quite prevalent in adolescent populations [13,15]. A prior study has shown that students of high school age tend to over-report height and under-report weight, therefore decreasing overweight and obesity prevalence estimates with lower average self-reported BMI [16]. Appropriate perception of weight is key in determining the nutritional habits and weight management of adolescents, it has been demonstrated that many students who are overweight are unlikely to participate in weight control practices [7,15,17,18]. The same work postulates that sex and race differences in weight perception suggest the need for tailored interventions for specific subpopulations and that as more adolescents become overweight, the comparison of oneself to peers may no longer lead to accurate weight perceptions of being overweight [15]. The following study aims to assess perceptions of overall health, weight status, and weight gain in relation to self-reported height and weight (BMI) among high school students in the state of Arkansas.

## Materials and methods

Study population

A validated survey-based cross-sectional study was conducted among 142 high school students in the state of Arkansas. This sample size was required to achieve the 80% power of the study. Students at Dardanelle High School enrolled in physical activity class during the fall 2018 semester were asked to voluntarily participate in the study. Students were given the survey along with a consent form to take home for completion. The sample included a total of 72 female high school students and 70 male high school students with an age range of 14 to 18 years old. The sample included 77 students in the 14-15-year age range and 65 students in the 16-18-year age range. The sample population was selected from grades nine through 12, which included 43 total freshmen, 49 total sophomores, 19 juniors, and 33 seniors that participated in the study. The sample population included three ethnic groups, White, Black, and Hispanic. The White population within the sample was disproportionately large composing 101 of the 142 total students. Self-reported height and weight measurements were used to calculate BMI (defined as weight in kilograms divided by the square of height in meters). Standard BMI cut points as defined by the CDC were used to place students into three major BMI categories, underweight (BMI <18.5), normal weight (BMI 18.5-24.99), and overweight/obese (BMI >25.0) [19]. A total of 11 students were classified as underweight, 57 students were classified as normal weight, and 74 students were classified as overweight/obese.

Arkansas Tech University IRB approved the project. Permission was also obtained from the principal of the high school.

Measures

A validated Student Obesity, Weight Loss, Physical Activity, and Nutrition survey was utilized to record the responses to a series of questions pertaining to self-perception of weight status, BMI status, and overall health. In regard to measuring self-perceived weight status, the following question was used: “Do you consider yourself now to be underweight, overweight, or about right?” Regarding evaluating self-perceived overall health, the following question was used: “In general, how would you rate your overall health?” Possible responses to the question include “excellent,” “very good,” “good,” “poor,” and “don’t know.” BMI was calculated based on self-reported height and weight; participants were then asked to classify their BMI as “overweight,” “underweight,” “normal weight,” or “don’t know.” Participants were also asked to rate their health and how much they worry about gaining weight.

Statistical analysis

Analysis of the data included cross-tabulation between self-perceived weight status and overall health perception, cross-tabulation between overall health perception and BMI perception, and cross-tabulation between self-perceived weight status and BMI perception. An ordered logistic regression model was then used to evaluate the response to the question on self-perceived weight status regarding age, year in school, race, and BMI. Confidence intervals of 95% were used for estimates throughout. Statistical analysis was performed using IBM SPSS Statistics for Windows, Version 26.0 (Released 2019, IBM Corp., Armonk, NY) [20].

## Results

Table 1 displays the demographic characteristics of the participants. There were 142 participants in this study, 72 females and 70 males ages 14-18 years old. Of the 142 participants, there were 43 freshmen, 49 sophomores, 19 juniors, and 31 seniors. Regarding race, there were 101 White students, six Black students, and 35 non-White Hispanic students. Table 2 displays a cross-tabulation of results for the question on self-perceived weight with self-perceived overall health status. With respect to self-perceived weight status; the majority of those in the “do not know” category perceive their overall health as “good” (7.2%), the majority of those in the “about right” category perceive their overall health as “good” (22.3%), the majority of those in the “overweight/obese” category perceive their overall health as “good” (15.8%), and the majority of those in the “underweight” category perceive their overall health as “good” (4.3%). Overall, with respect to the parameters of Table 2, most students (47.5%) perceive their weight status as “about right”. Discrepancies include the misclassification of perceived weight status of “overweight/obese” as either “good” or “very good” with respect to perception of overall health (c2= 11.352, p = 0.499).

**Table 1:** Sample Characteristics

	n (%)
Number of Participants	142 (100)
Gender	
Males	70 (49.3)
Females	72 (50.7)
Age (years)	
14-15	77 (54.2)
16-18	65 (45.8)
High School year	
Freshman	43 (30.3)
Sophomore	49 (34.5)
Junior	19 (13.4)
Senior	31 (21.8)
Race	
White	101 (71.1)
Black	6 (4.2)
Non-white Hispanic	35 (24.6)
Height mean (m)	1.76
Weight mean (kg)	80.42
BMI mean (kg/m^2^)	25.98
BMI category	
Underweight (<18.5)	11 (7.7)
Normal weight (18.5-24.99)	57 (40.1)
Overweight/obese (>25.0)	74 (52.1)

**Table 2:** Comparison of How Do You Consider Yourself Now with Overall Health Perception (ab) Pearson Chi-Square value = 11.352, df = 12, Significance = 0.499 a. Cell percentages (%) are weighted using survey sample weights; b. Consider yourself (in terms of body weight): Obese (BMI =30 or more); overweight (BMI =25.0-29.99); normal (BMI=18.5–24.9); underweight (BMI<18.5)

n=139	Perception of Overall Health				
Consider Yourself (In terms of body weight)	Do not know n (%)	Poor n (%)	Good n (%)	Very good n (%)	Excellent n (%)
Do not know	2 (1.4)	0	9 (7.2)	1 (0.7)	1 (0.7)
About Right	3 (2.2)	3 (2.2)	31 (22.3)	23 (16.5)	6 (4.3)
Overweight/Obese	3 (2.2)	6 (4.3)	22 (15.8)	13 (9.4)	2 (1.4)
Underweight	1 (0.7)	1 (0.7)	6 (4.3)	4 (2.9)	2 (1.4)

Table 3 displays a cross-tabulation of self-perceived overall health with self-perceived BMI. With respect to overall health; the majority of those in the “do not know” category perceive their BMI as “do not know” (2.1%) and “normal weight” (2.1%), the majority of those in the “poor” category perceive their BMI as “normal weight” (3.5%), the majority of those in the “good” category perceive their BMI as “overweight” (19.9%), the majority of those in the “very good” category perceive their BMI as “overweight” (13.5%), and the majority of those in the “excellent” category perceive their BMI as “overweight” (5%). Overall, with respect to the parameters of table 3, most students (48.9%) perceive their overall health as “good". Discrepancies include the misclassification of those with the perceived overall health of “good” or “very good” as “overweight” with respect to self-perceived BMI (c2= 20.321, p = 0.061).

**Table 3:** Comparison of Overall Health Perception with BMI Pearson Chi-Square value = 20.321, df = 12, Significance = 0.061 a. Cell percentages (%) are weighted using survey sample weights; b. Consider yourself (in terms of body weight): Obese (BMI =30 or more); overweight (BMI =25.0-29.99); normal (BMI=18.5–24.9); underweight (BMI<18.5)

n=141	BMI			
Overall Health	Do not know n (%)	Overweight n (%)	Normal Weight n (%)	Underweight n (%)
Do not know	3 (2.1)	1 (0.7)	3 (2.1)	2 (1.4)
Poor	1 (0.7)	1 (0.7)	5 (3.5)	3 (2.1)
Good	2 (1.4)	28 (19.9)	24 (17.0)	15 (10.6)
Very Good	3 (2.1)	19 (13.5)	11 (7.8)	8 (5.7)
Excellent	2 (1.4)	7 (5.0)	2 (1.4)	1 (0.7)

Table 4 displays a cross-tabulation of results for the question on self-perceived weight with self-perceived BMI. With respect to self-perceived weight status; the majority of those in the “do not know category” perceive their BMI as “do not know” (2.9%), “overweight” (2.9%), and “normal weight” (2.9%), the majority of those in the “about right category” perceive their BMI as “normal weight” (25.7%), the majority of those in the “overweight” category” perceive their BMI as “do not know” (15%), and the majority of those in the “underweight” category perceive their BIM as “normal weight” (6.4%). Overall, with respect to the parameters of table 4, most students (47.2%) perceive their weight status as “about right”. Discrepancies include the misclassification of perceived weight status of “about right” as “overweight” with respect to self-perceived BMI and misclassification of perceived weight status of “overweight” as “do not know” with respect to self-perceived BMI (c2 = 54.773, p = 0.000).

**Table 4:** Comparison of How Do You Consider Yourself Now with BMI (ab) Pearson Chi-Square value – 54.773, df = 9, Significance = 0.000 a. Cell percentages (%) are weighted using survey sample weights; b. Consider yourself (in terms of body weight): Obese (BMI=30 or more); overweight (BMI =25.0-29.99); normal (BMI=18.5–24.9); underweight (BMI<18.5)

n=140	BMI			
Consider Yourself (In terms of body weight)	Do not know n (%)	Overweight n (%)	Normal Weight n (%)	Underweight n (%)
Do not Know	4 (2.9)	4 (2.9)	4 (2.9)	1 (0.7)
About Right	4 (2.9)	21 (15)	36 (25.7)	5 (3.6)
Overweight	21 (15)	17 (12.1)	8 (5.7)	0
Underweight	0	1 (0.7)	9 (6.4)	5 (3.6)

Table 5 shows an ordered logistic regression model of response to the question on self-perceived weight. Students in the age range 14-15 years old are five times more likely than the 16-18-year-old age group to place themselves in a heavier and underweight category (p = 0.008, CI = 1.511-16.234). Regarding gender, males were 1.432 times more likely to place themselves into overweight and underweight categories relative to females. Regarding year in school, freshmen are 0.289 times less likely, sophomores 0.365 times less likely, and juniors 1.045 times more likely to place themselves in the overweight and underweight category relative to seniors. When considering race, non-White Hispanic students are 0.555 times less likely and Black students 0.835 times less likely to place themselves in the overweight and underweight category relative to White students. When considering BMI, those with an underweight BMI were four times more likely to place themselves in the overweight and underweight category relative to the normal weight BMI category (p = 0.032, CI = 1.131-14.216).

**Table 5:** Factors Associated with Consider Yourself in Terms of Body Weight (a) *p<0.05 a. The table shows an ordered logistic regression model of response to the question on self-perceived weight. OD refers to the odds of being in a higher (heavier) and underweight category rather than normal-weight perception category, with normal weight as the reference category. S: Significance value

	Consider Yourself		
	S	OR	CI
Age (years)			
14-15	0.008*	4.953	1.511 – 16.234
16-18		1	
Gender			
Male	0.276	1.432	0.750 – 2.733
Female		1	
Year in school			
Freshman	0.104	0.289	0.065 – 1.288
Sophomore	0.115	0.365	0.104 – 1.278
Junior	0.939	1.045	0.337 – 3.242
Senior		1	
Race			
Non-white Hispanic	0.135	0.555	0.256 – 1.202
Black	0.825	0.835	0.168 – 4.156
White		1	
BMI			
Underweight	0.032*	4.009	1.131 – 14.216
Overweight	0.691	1.168	0.543 – 2.511
Obese	0.051	2.369	0.998 – 5.625
Normal Weight		1	

## Discussion

The findings from cross-tabulation of results highlighted the inaccuracy in which high school-age students perceive their weight status, overall health, and BMI. An inaccurate understanding of these parameters increases the risk of being overweight/obese as well as decreases the likelihood that students will engage in healthy lifestyle behaviors such as adopting adequate nutrition plans and increasing physical activity.

Regarding demographics, two key findings were observed both of which were statistically significant. First, students 14-15 years old were observed to be five times more likely than older students to place themselves in a heavier and underweight category. This finding is statistically significant and reflects age differences in perception of weight, overall health, and BMI. Secondly, those with an underweight BMI were four times more likely to place themselves in the overweight and underweight category relative to the normal BMI category. This finding is also statistically significant and reflects that those with an underweight BMI are more likely to either classify their weight status correctly or significantly misclassify their weight status. Other key findings include differences in gender and race, although neither are statistically significant. Regarding gender, males were 1.432 times more likely to place themselves into the overweight and underweight categories relative to females. Previous research has demonstrated significant differences in weight perception between adolescent males and females, with females tending to be more likely to report feelings of body dissatisfaction in terms of weight [21,22]. A study conducted in Europe on university students demonstrated similar results, showing that females are more likely to consider themselves overweight at a normal BMI while males are more likely to consider themselves underweight at a normal BMI [23]. A total of 5900 university students across multiple European Universities were given a questionnaire and results showed “fairly uniform” outcomes regarding perceived weight status in regard to BMI across Europe, which are congruent with the results of this study [23]. Regarding race, non-White Hispanic students are 0.555 times less likely and Black students 0.835 times less likely to place themselves in the overweight and underweight category relative to White students. The findings regarding race reflect previous work, which has shown that White adolescents tend to be more likely to report body dissatisfaction in terms of weight. [21] It has also been shown in previous work that White adolescents compared to Black adolescents are more likely to engage in dieting behaviors as well as engagement in physical activity to lose weight [24]. Furthermore, White adolescent females have been observed to be twice as likely to perceive themselves as overweight compared to Black females. [24]

This study has important limitations to be considered. First, the sample size in this study includes 142 participants. It is quite clear that increasing the population size will lead to increased reliability of results and will better reflect the general population. Second, the population in this study was composed of 71.1% White students, 24.6% non-White Hispanic students, and only 4.2% Black students. The population included in the study does not accurately reflect race proportions in the US, therefore drawing conclusions about differences in race regarding weight perception, overall health, and BMI should be avoided. Third, BMI does not differentiate between muscle mass and fat mass. BMI therefore may inaccurately classify certain people as overweight when they simply have a larger muscle to fat ratio than average. BMI in this context is simply a proxy and utilizing more accurate body fat measurement would be ideal. Lastly, the BMI was calculated based on self-reported height and weight by the high school students, which could classify students into a different category than an actual category. 

Previous work has shown that major differences in weight control behavior exist between sex, race, and age [25]. Furthermore, it was observed that adolescents’ perception of being overweight or normal weight correlates poorly with medical definitions of actually being overweight [25]. Implementing successful programs that focus on behavior change and assessing willingness to change has been successful, but require effective communication between healthcare professional, the family, and the child [26]. These therapies should be implemented in a comprehensive fashion and in families that perceive obesity as an issue. [26]

Future directions include the additional investigation into the various complex social dynamics related to how adolescents determine self-perception of weight status. Investigating the various factors that adolescents value regarding how they perceive their body image and weight status will increase the efficacy of public health efforts in combating the obesity epidemic in adolescent populations around the globe. The challenge lies in developing such programs to deploy adequate public health initiatives aimed at going beyond simply educating adolescents about a healthy diet or lifestyle choices.

## Conclusions

The results of this study provide a framework for understanding the differences in how male and female high school students perceive their health, weight status, and weight gain in relation to BMI. Inaccurate perception of one’s weight status increases the risk of being overweight/obese and decreases the likelihood that students will engage in healthy lifestyle behaviors.
